# Modifiable environmental exposure and risk of rheumatoid arthritis—current evidence from genetic studies

**DOI:** 10.1186/s13075-020-02253-5

**Published:** 2020-06-22

**Authors:** Xia Jiang, Lars Alfredsson

**Affiliations:** 1 Department of Clinical Neuroscience, Center for Molecular Medicine, Karolinska Institutet, tomtebodavägen 18A, 5th floor, 171 77 Stockholm, Sweden; 2 Institute of Environmental Medicine, Karolinska Institutet, Stockholm, Nobels väg 13, 171 77 Sweden

**Keywords:** Rheumatoid arthritis, Modifiable environmental exposures, Genetic instruments, Mendelian randomization

## Abstract

Rheumatoid arthritis (RA) is a multifactorial chronic autoimmune disease, which involves a complex interplay of environmental triggers and genetic components in its etiology. It has been shown that genetics only explain about half of the liability to develop RA, leaving a large room for non-genetic factors. Indeed, several environmental exposures including smoking, drinking, obesity, and dietary patterns (and more) have been identified to be associated with RA risk, yet the observational nature of conventional epidemiological investigation hampers causal inference, as the validity of results could be plagued by measurement error, confounding, and/or reverse causality. Mendelian randomization (MR) is a novel statistical approach that uses genetic variants as instrumental variables (IV) to make causal inferences from observational data. The current genetic discoveries in the many heritable and modifiable human complex traits have provided an exceptional opportunity to evaluate a putative causal relationship between exposure and outcome in the absence of high-quality experimental or intervention studies, through a MR design. In the current review, we detail the contribution of MR studies hitherto conducted for modifiable environmental exposures with the risk of RA to understand the role of these factors in RA pathogenesis. We start with a brief introduction of each study, follow by a summarization of shortcomings and conclude by highlighting future directions. The application of MR design in the field of rheumatology remains limited. Only a few MR studies have examined the causal roles of vitamin D, cigarette smoking, alcohol consumption, coffee consumption, and levels of education in RA, where, no consistent evidence for a causal relationship has been found. Most studies lacked sensitivity analyses to verify MR model assumptions and to guarantee the validity of results. Almost all studies are likely to bias the strength of association towards a null value, since they used IVs from earlier GWAS(s) of exposures with a small sample size (i.e., few genetic markers). As the magnitudes of GWAS expand rapidly, additional trait-associated loci have been discovered. Incorporating these loci would greatly improve the strength of genetic instruments, as well as both the accuracy and precision of MR estimates. To conclude, there is a need for an update and a huge space for improvement of future MR studies in RA.

## Introduction

Rheumatoid arthritis (RA) is a chronic autoimmune inflammatory joint disease, which can progress to a declined functional status, severe comorbidity, and shortened lifespan if left untreated or poorly controlled. The disease is more prevalent in Nordic countries (affecting 1–2% of its population) and among women (with a female-to-male ratio of 2:1) [1–3].

As a multifactorial human disease, the etiology of RA involves a complex interplay of both environmental triggers and genetic components. A primary RA genetic risk factor is located within the major histocompatibility complex region (MHC) on chromosome 6, also known as the HLA shared epitope (HLA-SE) [4]. In addition, large-scale genome-wide association studies (GWAS) that aggregated existing genetic and biological data have identified more than 100 non-HLA loci associated with various immunological processes for the development of RA [5]. Despite these major genomic discoveries, the proportion of liability to develop RA that is due to genetic factors (i.e., the definition of heritability) has been estimated to be around 40–50% [6]. This estimate is consistent either by using classical pedigree-based design (familial or twin studies) or by direct quantification of genetic relatedness using genome-wide genetic markers (GCTA or Bayesian polygenic model). These figures indicate that environmental factors are likely to play a large role in RA etiology. Indeed, several environmental exposures including smoking, alcohol drinking, obesity, and dietary patterns (and more) have been observed to be associated with RA onset [7].

Unlike genetic findings, environmental factors are complicated to pinpoint. The observational nature of conventional epidemiological investigations (case-control or cohort design) which could confirm an exposure-outcome relationship usually hampers a causal interpretation, as the validity of results could be plagued by measurement error, confounding, and/or reverse causality. For example, many non-genetic modifiable factors including behavioral, socioeconomic, and physiological characteristics tend to occur in clusters—people with healthy diets often have other healthy habits [8]. Researchers conducting observational studies always try to adjust for confounding, but such adjustment will be hard to perform, partly because it will not always be clear which factors are confounders. Moreover, case-control studies may be limited by potential differential misclassification of the exposures through recall bias since cases often try to seek for a reason for their sickness and therefore tend to over report. Last but not least, reverse causation, which occurs when the disease status influences exposure rather than vice versa, is of particular concern in RA, as the onset of RA is often insidious and may precede the first clinical manifestation by several months to years, i.e., a rising level of antibodies can occur up to 7 years before diagnosis of ACPC-positive RA. Preclinical changes of RA (before a firm diagnosis can be verified) are likely to influence one’s lifestyle and yield to reverse causation if exposures are measured close to this stage. One such example has been raised in a likewise autoimmune disease, multiple sclerosis, where a significant association between having children and reduced MS risk has been identified, suggesting a protective effect of pregnancy. Yet the association was observed for both sexes and only within 5 years preceding MS diagnosis, raising a possibility of reverse causation wherein subclinical undiagnosed MS may have led to a decreased fecundity [9].

Largely due to these limitations, results from observational studies may fail to be replicated in randomized controlled trials (RCTs), the current gold standard for causal inference. Even though RCTs are widely recognized as the best possible solution, large-scale RCTs in RA are not currently prioritized or feasible due to their high cost and long duration. Certain environmental exposures such as smoking and alcohol drinking are not ethical to randomize. Nonetheless, even RCTs are likely to be underpowered given the low incidence of endpoint phenotypes (i.e., for RA, only 25 new patients can be identified following a population of 100,000 persons for an entire year).

Perhaps not surprisingly, most measures of the so-called “environment” show a significant and substantial genetic influence [10]. Encouragingly, the current genetic discoveries in several heritable and modifiable human complex traits (i.e., smoking behavior, alcohol consumption, body mass index, habitual physical activity, mood change) have provided an exceptional opportunity to evaluate a putative causal relationship between exposure(s) and outcome (RA) in the absence of high-quality experimental or intervention studies, that is, through Mendelian randomization (MR) design.

**Fig. 1:**
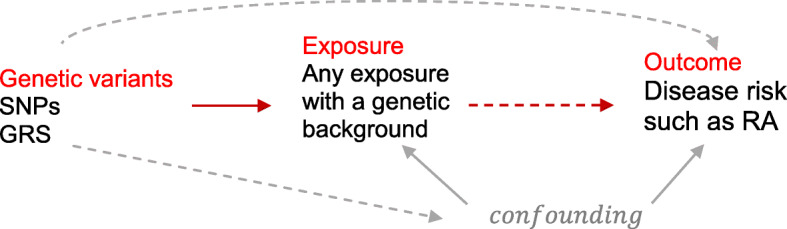
The conceptual framework of Mendelian Randomization. Mendelian randomization study uses genetic information (SNPs, instruments, IVs) as proxy for exposure to understand a causal inference between an exposure and an outcome. An un-confounded causal estimation can be made based on the observed IV-exposure and IV-outcome associations under certain assumptions. Namely, the selected IVs are associated with the exposure (red solid arrow), but not associated with any confounder of the exposure-outcome relationship (gray dash arrow), nor associated with the outcome via pathways other than through the exposure (gray dash arrow)

MR is an elegant tool and a novel statistical approach that uses genetic variants (single nucleotide polymorphisms, SNPs) as instrumental variables (IV) to make causal inferences from observational data. It is based on the fact that (I) SNPs (genotypes) are randomly assigned at conception, mirroring the randomization process in controlled trials and limiting the effect of confounding (at that stage), and (II) SNPs always precede disease onset, precluding reverse causality [11]. An un-confounded causal estimation can be made based on the observed IV-exposure and IV-outcome associations under certain assumptions. Namely, three important model assumptions need to be satisfied, that the selected IVs are associated with the exposure (*relevance*), but not associated with any confounder of the exposure-outcome relationship (*independence* or *exchangeability*), nor associated with the outcome via pathways other than through the exposure (*exclusion restriction*) (Fig. 1).

The emergence of MR as a powerful tool can be demonstrated by its rapid expansion in publications. A PubMed search for the terms “Mendelian randomization” or “Mendelian randomisation” identified only 98 papers in 2003 but 545 papers in 2019, a more than fivefold increased number of publications. Nonetheless, its utilization in rheumatology remains limited. Within the field of RA, 19 original MRs could be identified, of which, 12 explored modifiable environmental exposures (Table 1). In the following paragraphs, we detail the contribution of these studies to understand roles of modifiable environmental exposures in RA pathogenesis.

**Table 1:** Characteristics of the current Mendelian randomization studies conducted for modifiable environmental exposures and RA

Author	Year	Exposure				Outcome			Results
		Trait	Genetic data	Ancestry	#IV	Trait	Genetic data	Ancestry	
Bae and Lee	2018	Vitamin D	Summary statistics from two vitamin D GWAS. One consisted of 3538 individuals with replication in an additional 2151 individuals. The other consisted of 4501 persons from five cohorts.	Asian Indian + European	3	RA onset	Meta-GWAS of 5539 autoantibody-positive RA and 20,169 controls with replication in an independent set of 6768 RA and 8806 controls	European	No significant associations
Viatte et al.	2014	Vitamin D	SNPs in four vitamin D metabolism genes (GC, DHCR7/NADSYN1, CYP2R1 and CYP24A1) associated at genome-wide significance with circulating vitamin D levels.	European	20	RA radiological outcome	1433 patients with 2164 X-rays from the Norfolk Arthritis Register (NOAR) and in 443 RA patients with 2924 X-rays from the Early Rheumatoid Arthritis Study (ERAS)	European	No significant associations
Yarwood et al.	2013	Vitamin D	SNPs in three vitamin D metabolism genes (GC, DHCR7/NADSYN1, and CYP2R1) associated at genome-wide significance with circulating vitamin D levels.	European	6	RA treatment response	1396 RA patients from the Biologics in Rheumatoid Arthritis Genetics and Genomics Study Syndicate (BRAGGSS)	European	No significant associations
Bae and Lee	2018	Body mass index	Meta-GWAS for BMI in 322,154 individuals from the GIANT consortium.	European	68	RA onset	UK Biobank GWAS of 337,159 individuals (7480 RA cases and 329,679 controls)	European	Both IVW and median based methods showed evidence to support a causal association between BMI and RA
Qian et al.	2020	Smoking	The IV and the genetic association estimates for smoking initiation and lifetime smoking were obtained from a GWAS metaanalysis including 1,232,091 individuals and a GWAS of 462,690 individuals.	European	367, 124	RA onset	GWAS including 14,361 RA cases and 43,923 controls	European	Genetic predisposition to smoking initiation and lifetime smoking significantly increased RA risk
Bae and Lee	2019	Alcohol consumption	GWAS for alcohol intake frequency (increase) from 336,965 individuals included in the UK Biobank.	European	24	RA onset	Meta-GWAS of 5539 autoantibody-positive RA and 20,169 controls with replication in an independent set of 6768 RA and 8806 controls	European	No significant associations
Bae and Lee	2018	Coffee consumption	Summary data of meta-GWAS on coffee intake from 8 Caucasian cohorts (*n* = 18,176) and meta-GWAS of predominately regular-type coffee consumption (cups per day) among coffee consumers (*n* = 91,462).	European	4	RA onset	Meta-GWAS of 5539 autoantibody-positive RA and 20,169 controls with replication in an independent set of 6768 RA and 8806 controls	European	Genetic predisposition to coffee consumption significantly increased RA risk
Yuan et al.	2020	Iron	A large GWAS of 48,972 individuals, four measures, serum iron, serum transferrin saturation, ferritin, and transferrin.	European	5, 5, 6, 8	RA onset	Large-scale meta-analysis of over 20 GWASs including 14,361 RA cases and 43,923 controls	European	Consistent across four iron biomarkers, genetically high iron status was inversely associated with RA
Cheng et al.	2019	Mineral nutrients	Ca-related genetic variation was derived from 17 population-based GWAS (*n* = 39,400). The genetic variation of Mg is the result of a GWAS (*n* = 15,366) from the CHARGE Alliance. The Fe-related genetic variation was the GWAS result in 8 populations (*n* = 48,972). The genetic variation with Cu and Zn concentrations in erythrocytes were derived from the Queensland Institute of Medicine’s twins and their families (*n* = 2603).	Mixed population	8, 5, 14, 2, 3	RA onset	Meta-GWAS for 10 million RA-related SNPs were evaluated in a total of > 100,000 subjects of European and Asian ancestry (29,880 RA casesand 73,758 controls)	Asian and European	No consistent significant results between mineral nutrients and RA
Zhao et al.	2018	PUFA	Strong, independent genetic predictors of linoleic acid using the three most significant uncorrelated SNPs and seven uncorrelated SNPs in genes (FADS1, FADS2, and NTAN1) relevant to PUFA metabolism from a GWAS in 8631 adults.	European	10	RA onset	GWAS including 14,361 RA cases and 43,923 controls	European	Genetically instrumented LA was inversely associated with RA
Inamo	2019	Gut microbiome	GWASs for gut microbiome (totally 3326 individuals)	European	26	RA onset	GWAS including 14,361 RA cases and 43,923 controls	European	No significant associations
Bae and Lee	2018	Education	A UK Biobank GWAS (*n* = 293,723) on years of education	European	49	RA onset	Meta-GWAS of 5539 autoantibody-positive RA and 20,169 controls with replication in an independent set of 6768 RA and 8806 controls	European	The IVW method instructed an inverse causative relationship between years of education and RA

### Vitamin D

Vitamin D, as a fat-soluble vitamin and a steroid pre-hormone, is believed to possess an immune-modulatory effect [12]. Bae and Lee conducted the first two-sample (i.e., where the IV-exposure and the IV-outcome associations are from two sets of participants) MR for vitamin D and RA. They selected three genome-wide significant vitamin D-associated independent SNPs located in SSTR4 (rs2207173), GC (rs2282679), and NADSYN1 (3829251) as IVs and applied these IVs on information from a meta-GWAS of 5539 autoantibody-positive RA patients and 20,169 controls, all of European ancestry. They used several MR approaches including an inverse variance weight method (IVW), a weighted median method, and a MR-Egger regression. They did not find any evidence supporting a causal relationship between genetically predicted serum vitamin D concentrations and risk of RA (IVW: odds ratio (95% confidence interval) = 1.03 (0.91–1.16), *P* = 0.66; weighted median: OR (95%CI) = 1.03 (0.90–1.16), *P* = 0.70; MR-Egger: OR (95%CI) = 1.13 (0.87–1.46), *P* = 0.52) [13].

In addition to RA onset, Viatte et al. further examined 20 SNPs (rs6013897, rs17217119, rs10500804, rs1993116, rs10741657, rs7116978, rs127947147, rs127858787, rs12800438, rs4944076, rs4944997, rs7944926, rs4945008, rs3829251, rs2298850, rs3755967, rs2282679, rs1155563, rs17467825, rs7041) from four vitamin D metabolism genes (GC, DHCR7/NADSYN1, CYP2R1, and CYP24A1) with RA radiological outcome, as reflected by Larsen score and presence of erosion. The study was conducted in 1433 patients with 2164 X-rays from Norfolk Arthritis Register (NOAR) and in 443 RA patients with 2924 X-rays from Early Rheumatoid Arthritis Study (ERAS). Although serological status and HLA-SE showed consistent association with radiological outcomes in both cohorts, the vitamin D metabolism SNPs showed conflicting evidence. Only two SNPs at DHCR7 (rs4944997 and its perfect proxy rs4944076) presented a consistent effect size that achieved Bonferroni corrected level of significance after meta-analyzing NOAR and ERAS (*P* = 0.003 for rs4944997; *P* = 0.009 for rs4944076). Therefore, the authors concluded that the effect of vitamin D SNPs and consequently vitamin D levels is unlikely to play a major role in the etiology of RA radiographic damage [14].

Similarly, Yarwood et al. investigated the effect of vitamin D on treatment response, testing six vitamin D-associated SNPs (rs10741657, rs3829251, rs1790349, rs12785878, rs1155563, and rs7041 on genes GC, DHCR7/NADSYN1, and CYP2R1) for association with response to anti-TNF therapy, in 1396 RA patients from Biologics in Rheumatoid Arthritis Genetics and Genomics Study Syndicate (BRAGGSS). Only one SNP showed modest association with absolute changes in DAS28 (*P* = 0.04 for rs10741657) yet did not withstand the Bonferroni correction, and no SNP was associated with good versus poor EULAR response. These results indicate a null causal link between genetically predicted vitamin D (using those SNPs) and RA treatment response [15].

### Obesity

Adipose tissue secretes pro-inflammatory cytokines, hormonally active substances, and chemokines and is therefore generally considered to be actively involved in immunity [16]. Bae and Lee investigated whether excessive body weight as measured by body mass index (BMI) could causally increase the risk of RA. The authors used publicly available summary statistics of a BMI GWAS conducted by the GIANT consortium in 322,154 individuals of European ancestry as exposure and a GWAS of self-reported RA among 337,159 individuals (RA case = 7480 and control = 329,679) included in UK Biobank as outcome, and selected 68 genome-wide significant BMI-associated SNPs as IVs. The IVW method showed evidence for a causal association where per unit increment in the genetically predicted BMI significantly increased RA risk (OR (95%CI) = 1.003 (1.001–1.004), *P* = 0.03). The effect remained of similar magnitude in weighted median approach (OR (95%CI) = 1.006 (1.002–1.01), *P* = 0.004), but attenuated to borderline significance in MR-Egger regression (OR (95%CI) = 1.004 (1.00–1.01)). These results suggest that genetic predisposition to obesity may play an important role in the development of RA [17].

### Smoking

Smoking is a primary environmental risk factor of RA. As an inhalable airborne exposure, smoking may trigger RA-specific immune reactions to citrullinated proteins in particular among individuals who are genetically susceptible to RA [18]. Qian et al. applied MR approach to examine the potential causal relationship between smoking and risk of RA. They obtained summary statistics data for RA from a meta-GWAS including 14,361 RA cases and 43,923 controls of European ancestry. The IVs and genetic associations of two smoking-related measures, smoking initiation (never/ever) and lifetime smoking (capturing smoking duration, heaviness, and cessation), were obtained from a meta-GWAS including 1,232,091 individuals and a meta-GWAS of 462,690 individuals of European ancestry. The authors found that compared with never smokers, genetic predisposition to smoking initiation was positively associated with risk of RA (IVW: OR (95%CI) = 1.32 (1.15–1.52), *P* = 9.17 × 10^−5^). Similarly, genetically predicted lifetime smoking was also found to be associated with an increased risk of RA (OR (95%CI) = 1.55 (1.13–2.14), *P* = 0.007). Sensitivity analyses using alternative MR methods and different sets of IVs produced similar results, suggesting the robustness of findings [19].

### Alcohol consumption

Alcohol contains components such as ethanol and antioxidants, which suppresses immune responses and reduces the synthesis of pro-inflammatory cytokines. Alcohol drinking is found to be associated with a decreased risk of RA from observational studies [20]. Bae and Lee examined whether alcohol intake is causally associated with RA by a two-sample MR. They used publicly available summary statistics of alcohol intake frequency from a UK Biobank GWAS (*n* = 336,965) as exposure and a meta-GWAS of 5539 autoantibody-positive RA patients and 20,169 controls as outcome. They selected 24 alcohol-associated genome-wide significant SNPs as IVs to improve inference. Although 16 IVs were inversely associated with RA, the IVW method showed no evidence of a causal relationship (OR (95%CI) = 1.24 (0.82–1.89), *P* = 0.31). The weighted median method (OR (95%CI) = 0.75 (0.42–1.36), *P* = 0.34) and MR-Egger regression (OR (95%CI) = 0.46 (0.07–2.94), *P* = 0.42) revealed similar null findings [21].

### Coffee consumption

Coffee, as one of the most commonly consumed beverages, contains a complex blend of bioactive compounds, exerting different physiological effects. Caffeine, for example, besides being the most frequently ingested psychoactive molecule, has important anti-apoptotic effects. Lipid molecules, like cafestol and kahweol, and antioxidant substances such as polyphenols have an indispensable role in scavenging free radicals as well as in inducing the activation of DNA repair and detoxification enzymes [22]. Bae and Lee analyzed the causal association between coffee consumption and risk of RA through a two-sample MR. Four genome-wide significant SNPs associated with regular coffee consumption (cups per day) were selected as IVs: NCARD (rs16868941), POR (rs17685), CYP1A1 (rs2470893), and LAMB4 (rs382140). Both IVW and weighted median methods showed a causal association between coffee consumption and RA (OR (95%CI) = 2.16 (1.25–3.73), *P* = 0.006; 2.12 (1.07–4.19), *P* = 0.03) while the effect attenuated to null in MR-Egger regression (*P* = 0.355). Noteworthy, the weak instrument (only 4 IVs collectively explained a small proportion of phenotypic variance) may bias the causal estimate [23].

### Mineral nutrition, PUFA, and microbiome

Mineral nutrients such as iron are important for human health through its vital role in oxygen transport, DNA biosynthesis, and energy metabolism and may cause disease if out of balance. Yuan et al. conducted a two-sample MR to assess the association of iron homeostasis with the risk of RA. Twelve SNPs associated with iron status at genome-wide significance level were selected from a large GWAS of 48,972 European-descent individuals. The author found that genetic predisposition to high iron status was causally associated with lower odds of RA (IVW: OR (95%CI) = 0.79 (0.65–0.94), *P* = 0.010; 0.59 (0.40–0.86), *P* = 0.007; 0.84 (0.75–0.94), *P* = 0.003 and 1.28 (1.06–1.55), *P* = 0.012 per one standard deviation increment of serum iron, ferritin, transferrin saturation, and transferrin levels, respectively). However, a separate MR study expanding the analysis to additional micronutrients including calcium, magnesium, copper, and zinc did not identify a consistent causal association for these elements and RA [24].

In addition to mineral nutrients, two other studies examined the roles of n-6 polyunsaturated fatty acid (PUFA) and microbiome in RA onset. Linoleic acid (LA), as a major PUFA, stimulates the synthesis of testosterone in animal models and may influence the risk of RA. Zhao et al. obtained strong, independent genetic predictors of LA using 10 SNPs in genes (FADS1, FADS2, and NTAN1) relevant to PUFA metabolism from a GWAS in 8631 adults of European ancestry. They found that genetically instrumented LA was inversely associated with RA (OR (95%CI) = 0.97 (0.95–0.98)) [25]. Moreover, immune responses in gut due to microbiome imbalance or maladaptation (intestinal dysbiosis) may also trigger the development of RA. Ianmo et al. examined 26 SNPs from a gut microbiome GWAS yet did not find convincing evidence for a causal link (*p* values derived from IVW, MR-Egger, and weighted median methods were not significant, *P* = 0.286, 0.057 and 0.166), suggesting that dysbiosis might be secondary phenomenon rather than triggers in the pathogenesis of RA [26].

### Education

Educational attainment is a simple measurable proxy for socioeconomic status. Bae and Lee chose summary genetic data on years of education from the UK Biobank GWAS of 293,723 individuals as the exposure and a meta-GWAS with autoantibody-positive RA cases (*n* = 5539) and European controls (*n* = 20,169) as the outcome. They selected a total of 49 SNPs as IVs. It might not be surprising that the IVW method instructed an inverse causative relationship between years of education and RA (OR (95%CI) = 0.47 (0.27–0.82), *P* = 0.008). The beneficial effect is more likely to be mediated through other healthy behaviors rather than education itself posing a direct effect on the etiology of RA; thus, sensitivity analysis is required to validate the MR model assumptions [27].

### Others

In addition to making causal inference between exposure and disease, MR can also contribute to the development of new treatments through analysis of genetic variations within drug target loci. For example, an association between a genetic polymorphism of interleukin-6 receptor (IL-6R) and risk of coronary heart disease has led to RCTs of tocilizumab, an IL-6R inhibitor (also an effective biological disease-modifying anti-rheumatic drug DMARD), in myocardial infarction [28]. Similarly in RA, Prins et al. performed an MR study using two genetic risk scores (GRS) of C-reactive protein (CRP) as IVs. The first GRS consisted of four SNPs in the CRP gene, and the second consisted of 18 SNPs that were significantly associated with CRP levels in a large GWAS of > 80,000 individuals. They found that genetically elevated CRP levels showed a significant potentially protective causal relationship with risk of RA after correction for heterogeneity. This implies that CRP-lowering interventions may not be likely to result in a decreased risk for a complex disease as RA [29]. Similar analyses have been conducted for other biomarkers such as glycosylation of IgG [30] and telomere length [31]; both provided novel insights into RA treatment strategy.

## Summarization, conclusion, and future direction

Modifiable environmental exposures lend themselves well to intervention and MR is an elegant genetic tool to inform intervention for improving public health. Leveraging large-scale genome-wide genetic data, results from MR studies provides a complement to the conventional epidemiological setting. They also provide novel insights into the mechanistic developmental processes of RA, as well as a list of actionable strategies that might mitigate RA risk. However, as the methodology continues to evolve and the genetic data continues to accumulate, it leaves a huge space for improvement of future MR designs in RA or in other chronic complex human diseases of similar kind. We, hereby, conclude by summarizing drawbacks underlying the current MR studies, providing solutions and highlighting future directions.

First of all, for MR results to be valid, three important model assumptions need to be satisfied, namely that the selected IVs are associated with exposure (relevance), but not associated with any confounder of the exposure-outcome relationship (independence or exchangeability), nor associated with the outcome via pathways other than through the exposure (exclusion restriction). While the first assumption is naturally met by using exposure-associated GWAS-identified variants, violation of the other two assumptions needs additional analyses to detect. Several important sensitivity analyses should be performed to guarantee model assumption. For example, MR-Egger regression [32] and MR-PRESSO [33] detect and correct for bias due to horizontal pleiotropy, where the average of direct effects of tested genetic variants on outcome is non-zero (i.e., violation of exclusion restriction assumption). In addition, analysis should be performed excluding SNPs shown to be associated with potential confounders of the exposure-outcome association according to GWAS catalog (i.e., violation of independence assumption). Moreover, leave-one-out analysis identifies potential influence of outlying variants on the estimates, and multivariable MR approach can be used to adjust for potential horizontal pleiotropy acting through certain variables. For example, the effect of lifestyle factors including smoking, drinking, and educational attainment is likely to act through body mass index. A detailed description of solutions to satisfy the three core MR assumptions as well as to deal with potential pitfalls has been reviewed by Dr. Zheng et al. [34] To the best of our knowledge, not all aforementioned MR studies have strictly verified the three model assumptions to guarantee the validity of results and therefore might yield to biased estimates.

Noteworthily, despite the many solutions proposed for satisfying MR model assumptions, these falsification strategies can only detect that an assumption is violated but cannot ever confirm that it holds. For example, current identification of IV-exposure associations as well as IV-confounder associations relies heavily on conventional techniques (genome-wide scan) and established knowledge. Even the hitherto largest GWAS does not disclose a full list of IVs for a given exposure. Through literature review and GWAS catalog look-up, we could probably confirm that an exposure-associated SNP is *so far* only reported to be associated with that particular exposure, but we can never guarantee the same SNP not to be associated with other traits (confounders)—it might be that the association remains to be identified, or it might be that the SNP is associated with an underlying risk factor that is unrecognized (e.g., this can be applied to many binary diseases which researchers believe an underlying latent continuous variable can be hypothesized behind a binary outcome). Moreover, small proportion of phenotypic variance explained by GWAS-discovered SNPs and unknown confounders remain two major challenges to be solved, which current MR can hardly control.

Secondly, for two-sample MR to be valid, exposure and outcome samples have to be from the same underlying population. Therefore, if IV-exposure associations are estimated based on a European population, an ideal outcome population should also be of the same ancestry. However, in several of the abovementioned MR studies, the authors used GWAS estimates of a European population as exposure but GWAS estimates of an admixed population (European + Asian) as outcome, which might introduce bias arisen from population stratification. It is very likely that the effect of a genetic variant from a combined mixed population not any close to its true effect in either of the subpopulations.

Furthermore, most of the MR studies examined by our current review used IVs from older GWAS(s) of exposures with smaller sample sizes and were therefore likely to be underpowered. As sample size of GWAS expands rapidly, additional trait-associated loci have been recently discovered (Table 2). For example, the most updated vitamin D GWAS has been conducted in a UK Biobank sample of 417,580 Europeans and revealed 143 independent loci (as compared to the 20 SNPs or fewer used by Viatte et al., Bae and Lee, and Yarwood et al.). The most updated alcohol GWAS has been conducted in sample of up to 1.2 million individuals and identified 99 SNPs for drinks per week (as compared to the 24 SNPs used by Bae et al.). The genetic architecture of exposures associated with multiple stages of tobacco use (initiation, cessation and heaviness) has also been examined. Incorporating these loci could greatly improve the strength of genetic instruments, as well as both the accuracy and precision of MR estimates.

**Table 2:** Current progress in genetic discoveries for some of the modifiable environmental risk factors

Traits	Author	Measures	Ancestry	Year	# Individuals	# IVs	Variance explained by genome-wide SNPs
Smoking	Liu et al.	Smoking initiation (ever vs. never smoking)	European	2019	1,232,091	378	8.0%
Smoking	Liu et al.	Cigarette per day	European	2019	337,334	55	7.8%
Smoking	Liu et al.	Smoking cessation	European	2019	547,219	24	4.6%
Alcohol consumption	Liu et al.	Drinks per week	European	2019	941,280	99	4.2%
Physical activity	Doherty et al.	Accelerometer-measured overall physical activity	European	2018	91,105	14	15%
Physical activity	Klimentidis et al.	Self-reported habitual physical activity	European	2018	337,234	8	5%
Body mass index	Pulit et al.	Body mass index	European	2018	694,649	941	22.4%
Vitamin D	Revez et al.	Circulating vitamin D levels	European	2019	417,580	143	13%
Vitamin D	Manousaki et al.	Circulating vitamin D levels	European	2020	401,460	138	4.9%
Educational attainment	Lee et al.	Number of years of schooling	European	2018	1,131,881	1271	12.2%
Depression	Howard et al.	Major depression disorder	European	2019	807,553	102	8.9%
Mood change	Ward et al.	Mood instability	European	2019	363,705	46	9.0%

Last but not least, to the best of our knowledge, no sex-specific analysis has been currently performed to understand sex disparity underlying the disease, despite RA strikes women 2–3 times more often than men. Most GWAS(s) include a large proportion of women (48–100%), yet sex-specific effect estimates are reported still only for a minority of GWAS. Leveraging the available sex-specific genetic data to perform analysis for men and women separately would be an important future direction to focus on. Efforts should also be made to increase generalizability as almost all current MR studies in RA are conducted in European populations. Future MR analysis should also be carried out to explore treatment strategy and prognosis of RA in addition to disease onset.

## Data Availability

Not applicable.

## Abbreviations

GWAS: Genome-wide association study
MR: Mendelian randomization
RA: Rheumatoid arthritis
OR: Odds ratio
CI: Confidence interval
IVW: Inverse variance weighted
SNP: Single nucleotide polymorphism
IV: Instrumental variable

## References

[1] Klareskog L, Catrina AI, Paget S (2009). Rheumatoid arthritis. Lancet Lond Engl..

[2] Lee DM, Weinblatt ME (2001). Rheumatoid arthritis. Lancet Lond Engl.

[3] Scott DL, Wolfe F, Huizinga TWJ (2010). Rheumatoid arthritis. Lancet Lond Engl..

[4] Holoshitz J (2010). The rheumatoid arthritis HLA-DRB1 shared epitope. Curr Opin Rheumatol.

[5] Okada Y, Eyre S, Suzuki A, Kochi Y, Yamamoto K (2019). Genetics of rheumatoid arthritis: 2018 status. Ann Rheum Dis.

[6] Frisell T, Saevarsdottir S, Askling J (2016). Family history of rheumatoid arthritis: an old concept with new developments. Nat Rev Rheumatol.

[7] Jiang X, Frisell T, Askling J, Karlson EW, Klareskog L, Alfredsson L (2015). To what extent is the familial risk of rheumatoid arthritis explained by established rheumatoid arthritis risk factors?. Arthritis Rheumatol.

[8] Smith GD, Lawlor DA, Harbord R, Timpson N, Day I, Ebrahim S (2007). Clustered environments and randomized genes: a fundamental distinction between conventional and genetic epidemiology. PLoS Med.

[9] Hedström AK, Hillert J, Olsson T, Alfredsson L (2014). Reverse causality behind the association between reproductive history and MS. Mult Scler Houndmills Basingstoke Engl.

[10] Plomin R, DeFries JC, Knopik VS, Neiderhiser JM (2016). Top 10 replicated findings from behavioral genetics. Perspect Psychol Sci J Assoc Psychol Sci.

[11] Smith GD, Ebrahim S (2003). “Mendelian randomization”: can genetic epidemiology contribute to understanding environmental determinants of disease?. Int J Epidemiol.

[12] Jiang X, Kiel DP, Kraft P (2019). The genetics of vitamin D. Bone..

[13] Bae S-C, Lee YH (2018). Vitamin D level and risk of systemic lupus erythematosus and rheumatoid arthritis: a Mendelian randomization. Clin Rheumatol.

[14] Viatte S, Yarwood A, McAllister K, Al-Mudhaffer S, Fu B, Flynn E (2014). The role of genetic polymorphisms regulating vitamin D levels in rheumatoid arthritis outcome: a Mendelian randomisation approach. Ann Rheum Dis.

[15] Yarwood A, Viatte S, Plant D, Morgan AW, Isaacs J, Wilson AG (2014). Testing the role of vitamin D in response to antitumour necrosis factor α therapy in a UK cohort: a Mendelian randomisation approach. Ann Rheum Dis.

[16] Gremese E, Tolusso B, Gigante MR, Ferraccioli G (2014). Obesity as a risk and severity factor in rheumatic diseases (autoimmune chronic inflammatory diseases). Front Immunol.

[17] Bae S-C, Lee YH (2019). Causal association between body mass index and risk of rheumatoid arthritis: a Mendelian randomization study. Eur J Clin Investig.

[18] Klareskog L, Malmström V, Lundberg K, Padyukov L, Alfredsson L (2011). Smoking, citrullination and genetic variability in the immunopathogenesis of rheumatoid arthritis. Semin Immunol.

[19] Qian Y, Zhang L, Wu DJH, Xie Z, Wen C, Mao Y (2020). Genetic predisposition to smoking is associated with risk of rheumatoid arthritis: a Mendelian randomization study. Arthritis Res Ther.

[20] Jin Z, Xiang C, Cai Q, Wei X, He J (2014). Alcohol consumption as a preventive factor for developing rheumatoid arthritis: a dose-response meta-analysis of prospective studies. Ann Rheum Dis.

[21] Bae S-C, Lee YH (2019). Alcohol intake and risk of rheumatoid arthritis: a Mendelian randomization study. Z Rheumatol.

[22] Sharif K, Watad A, Bragazzi NL, Adawi M, Amital H, Shoenfeld Y (2017). Coffee and autoimmunity: more than a mere hot beverage!. Autoimmun Rev.

[23] Bae S-C, Lee YH (2018). Coffee consumption and the risk of rheumatoid arthritis and systemic lupus erythematosus: a Mendelian randomization study. Clin Rheumatol.

[24] Yuan S, Larsson S. Causal associations of iron status with gout and rheumatoid arthritis, but not with inflammatory bowel disease. Clin Nutr. 2020;S0261-5614(20)30042-X. 10.1016/j.clnu.2020.01.019 32044136

[25] Zhao JV, Schooling CM (2019). Role of linoleic acid in autoimmune disorders: a Mendelian randomisation study. Ann Rheum Dis.

[26] Inamo J. Non-causal association of gut microbiome on the risk of rheumatoid arthritis: a Mendelian randomisation study. Ann Rheum Dis. 2019;annrheumdis-2019-216565. 10.1136/annrheumdis-2019-216565 31744823

[27] Bae S-C, Lee YH (2019). Causal relationship between years of education and the occurrence of rheumatoid arthritis. Postgrad Med J.

[28] Swerdlow DI, Holmes MV, Kuchenbaecker KB, JEL E, Shah T, Interleukin-6 Receptor Mendelian Randomisation Analysis (IL6R MR) Consortium (2012). The interleukin-6 receptor as a target for prevention of coronary heart disease: a mendelian randomisation analysis. Lancet Lond Engl.

[29] Prins BP, Abbasi A, Wong A, Vaez A, Nolte I, Franceschini N (2016). Investigating the causal relationship of C-reactive protein with 32 complex somatic and psychiatric outcomes: a large-scale cross-consortium Mendelian randomization study. PLoS Med.

[30] Yarwood A, Viatte S, Okada Y, Plenge R, Yamamoto K, BRAGGSS, RACI (2016). Loci associated with N-glycosylation of human IgG are not associated with rheumatoid arthritis: a Mendelian randomisation study. Ann Rheum Dis.

[31] Zeng Z, Zhang W, Qian Y, et al. Association of telomere length with risk of rheumatoid arthritis: a meta-analysis and Mendelian randomization. Rheumatology (Oxford). 2020;59(5):940-947. 10.1093/rheumatology/kez524 31697380

[32] Bowden J, Davey Smith G, Burgess S (2015). Mendelian randomization with invalid instruments: effect estimation and bias detection through Egger regression. Int J Epidemiol.

[33] Verbanck M, Chen C-Y, Neale B, Do R (2018). Detection of widespread horizontal pleiotropy in causal relationships inferred from Mendelian randomization between complex traits and diseases. Nat Genet.

[34] Zheng J, Baird D, Borges M-C, Bowden J, Hemani G, Haycock P (2017). Recent developments in Mendelian randomization studies. Curr Epidemiol Rep.

